# Impact of bottle size on in-home consumption of wine: feasibility and acceptability randomised cross-over study

**DOI:** 10.1186/s40814-020-00566-5

**Published:** 2020-02-11

**Authors:** Eleni Mantzari, Catherine Galloway, Gareth Hollands, Rachel Pechey, Zorana Zupan, Mark Pilling, Theresa Marteau

**Affiliations:** Behaviour and Health Research Unit, University of Cambridge, Forvie Site, Robinson Way, Cambridge, CB2 0SR UK

**Keywords:** Alcohol, Wine, Consumption, Bottle size, Portion size

## Abstract

**Background:**

Reducing alcohol consumption across populations would prevent many non-communicable diseases. Large packages increase food and non-alcoholic drink consumption and large glasses increase wine consumption. Smaller bottles may reduce alcohol consumption but their impact is uncertain. This study aims to (i) explore the feasibility and acceptability of conducting a large-scale randomised study to assess the impact of bottle size on in-home wine consumption and (ii) estimate the effect size and variance of the intervention on consumption to inform the design of future studies.

**Methods:**

Cross-over randomised study in which 16 households in Cambridge, England, consuming at least two 750-ml bottles of wine each week, received a pre-set volume of wine biweekly for 4 weeks, in 750-ml and 375-ml bottles, in random order. Consumption was assessed by recording the number of empty and partially full bottles at the end of each biweekly period. At the end of the study, household representatives were interviewed about their experiences of participating in the study.

**Results:**

The study procedures proved feasible. Comparable to similar trials, 14% of identified eligible households (18/125) consented to participate in the study. Attrition between consent and study completion was 11% (2/18) and 0% between study periods and 13% of households (2/16) correctly identified the study aim. The study procedures were considered acceptable. After adjusting for guest and out-of-home consumption, the difference in consumption between the 750-ml (3385.2 ml; SD = 1698.5) and 375-ml bottles (3376.7 ml; SD = 1719.0) was 8.4 ml (SD = 1235.4; 95%CI − 596.9, 613.8). Results suggest a possible order effect, with households receiving the 375-ml bottles first consuming more wine out of the 750-ml bottles and vice versa. This might also reflect an increase in consumption with study duration. Households receiving the 375-ml bottles first (6315.9 ml; SD = 3293.5) also drank less wine overall than those receiving the 750-ml bottles first (7335.4 ml; SD = 3735.4).

**Discussion:**

The findings support the feasibility and acceptability of running a large-scale randomised study to assess the impact of bottle size on in-home wine consumption. Due to the heterogeneous patterning of results, a future study will be powered using the variance observed in the current study to detect a meaningful reduction of 250 ml of wine when consumed from smaller compared with larger bottles.

**Trial registration:**

Open Science Framework (OSF): rmk43; May 23, 2017.

## Background

Alcohol consumption is the fifth leading cause of death and disability globally [1] and is linked to the development of non-communicable diseases, including some cancers, cardiovascular diseases and diabetes [2]. In 2017 in Great Britain, 57% of adults reported drinking alcohol in the previous week, 45% of whom consumed more than a third of their weekly unit allowance (14 units) on their heaviest drinking day, and 9% exceeded this limit [3]. In 2016 in the UK, 7327 deaths and more than one million hospital admissions were related to alcohol consumption [4, 5].

Contextual factors, including price and affordability, availability and marketing, may encourage excessive alcohol consumption [6–9]. Recently, the role of other factors, such as container and portion size, has also been highlighted. A Cochrane systematic review found that large portions and packages increase the consumption of food and non-alcoholic drinks [10]. Although this review did not identify any studies focusing on alcohol consumption, its findings imply that reducing the size of the containers in which alcohol is presented might be a promising intervention for reducing consumption. In line with this, recent field studies have shown that the size of glasses in which wine is served, while keeping the amount constant, can affect consumption, with larger glasses increasing consumption, particularly in restaurants [11–14].

Internationally, the most popular wine packaging format is the 750-ml bottle [15]. In recent years, some UK supermarkets have taken the initiative to sell non-premium wines in smaller bottles, in an effort to expand their sales, by providing a product that appeals to consumers wanting to consume less wine [16–18]**.** This follows the growth of wine drinking, with wine now being the most popular alcoholic drink for adults across all ages and regions of the UK. About 85% of wine in Great Britain is bought in off-licence outlets—mainly supermarkets—to consume at home, rather than in pubs and restaurants [3, 19]. Based on the aforementioned Cochrane review [10] and field studies [11, 12], it is expected that consuming from bottles smaller than 750 ml would reduce wine consumption. Given the lack of relevant evidence, however, uncertainty surrounds this hypothesis. In theory, smaller bottles have the potential to both decrease and increase consumption. They may decrease consumption through one of several mechanisms: making additional intake of wine more effortful, through the need to acquire and open multiple bottles [10], or as a result of individuals’ tendency to consume a specific number of bottles in any one episode of consumption regardless of bottle size, referred to as the “unit bias heuristic” [20]. Smaller bottles could also increase consumption through one of several mechanisms. First, smaller sized bottles may reduce barriers to consumption that are present for larger sizes. For example, small packages of crisps have been shown to inhibit concerns of overconsumption evoked by large packages of crisps [21]. Second, as 750-ml wine bottles have become the standard size for wine internationally, the amount of wine held in smaller bottles may be perceived to be too small. This may lead to consumption beyond the amount consumed when wine is presented in larger bottles [22], by encouraging consumption of multiple bottles during a consumption episode and—if offered in bulk—by increasing the frequency of consumption [23, 24].

In conclusion, there is an absence of evidence regarding the impact of bottle size upon wine consumption. In preparation for a study to generate such evidence, the current study aims to reduce key uncertainties related to its design.

### Aim and objectives

The aims of the current study are to assess the feasibility and acceptability of the procedures for a study designed to estimate the impact of bottle size upon in-home wine consumption and to provide data as the basis for a sample size estimate for the planned study.

The specific objectives are to describe and assess the following:

1. Feasibility
  - Feasibility of recruiting participants from eligible households into the study, and estimate recruitment and retention rates
  - Feasibility of delivering the intervention
  - Feasibility of the assessment procedures
  - Feasibility of collecting consumption-related data
  - Credibility of the study cover story and awareness of the purpose of the intervention
2. Acceptability
  - Acceptability of the intervention and study procedures
3. Sample size estimation
  - Possible effect size and variance, to inform sample size calculations for the planned study

## Methods

The study was pre-registered with the Open Science Framework (https://osf.io/rmk43/).

### Setting

The study was conducted in a community setting, comprising residential households in Cambridgeshire, England.

### Design

The study involved a cross-over design, without a washout period, in which general population households were each exposed to two intervention conditions over time, randomised in their order of presentation.

### Participants

Participants were 16 households with the following characteristics:

1. Included one or more adults, who together consumed wine at a minimum rate of 1500 ml per week (i.e. 2 × 750-ml bottles).
2. Were located in Cambridgeshire, England, within a 20-mile-radius of the research team base.
3. None of the adult wine drinkers planned to be away from home for longer than 7 days during the study period.
4. None of the adult wine drinkers took medications that can interact with alcohol (e.g. some antibiotics, sleeping tablets, opioid analgesics).
5. None of the adult wine drinkers had a history of becoming seriously ill (i.e. requiring hospitalisation) after alcohol consumption.
6. None of the adult wine drinkers had a history of alcoholism and/or severe mental health illnesses (e.g. paranoid and other psychotic disorders, bipolar disorders and schizoaffective disorders).

One adult from each eligible household was recruited to act as a household representative, who consented to participation in the study for the entire household and provided all necessary data. Households were identified and recruited through a research agency (Wyman Dillon, http://wymandillon.co.uk). A recruiter, employed by the researcher agency, approached individuals outside large retail stores in the target areas and assessed interest in the study and eligibility. The contact details of individuals belonging to eligible households, who were interested in taking part in the study, were passed onto the research team. Apart from the recruitment, the research agency did not conduct any other parts of the study.

### Intervention

The two intervention conditions comprised receipt of a given quantity of a wine from one of two differently sized bottles:

1. 750-ml bottles
2. 375-ml bottles

The bottle design (i.e. shape, colour and label) of each wine was identical in the two sizes. All participating households received both interventions according to a pre-specified random order (see the “Randomisation” section for details). Each intervention lasted two weeks. During the intervention weeks, households received their preferred wine(s), chosen from the study wine list, in just one bottle size. The wine list was compiled on the basis of wine that was available in both target bottle sizes and included a range of options from multiple regions, to cater to most preferences and budgets. The number of bottles supplied to households was determined by the total volume of wine each household received, which was fixed across the two intervention periods. The amount received was determined with reference to the volume of wine households typically consumed per week, rounded up to the nearest standard bottle and up to a maximum of 14 standard bottles (10,500 ml). The amount of each type of wine received was also kept constant during intervention periods and was determined based on consumption during baseline (used to determined amount of red vs white) and through discussion with household representatives. During each intervention fortnight, households were given the opportunity to receive additional deliveries if needed. The intervention can be categorised as a size × product intervention within the TIPPME intervention typology [25].

All wine deliveries were organised and completed by a trained researcher (CG). All wine used in this study was ordered and paid for by a leisure centre in Cambridge. The researcher conducting the study, who was authorised by the Designated Premise Supervisor to sell alcohol on behalf of the leisure centre, picked up the wine, delivered it to participating households and accepted payments for the wine consumed.

### Randomisation

The unit of randomisation was the household. Blocked randomisation was used to ensure that approximately equal numbers of households received each of the two bottle sizes during each intervention period. The randomisation was determined during a “run-in” period by a member of the research team (EM).

### Procedure

Participation comprised six stages.

#### Stage 1: Recruitment and baseline assessment period—2 weeks

During a recruitment visit with household representatives (i.e. individuals who were recruited from each household to provide the necessary data), conducted by the trained researcher, participants were given detailed information about the study and asked to give written informed consent on behalf of their household for participating in the study and for adhering to the study procedures. The study was presented as an investigation of the impact on the sensory experience of consuming wine from different-sized bottles. Specifically, household representatives viewed written information detailing that the study would explore whether different bottle sizes influence (1) taste, level of enjoyment and satisfaction associated with drinking wine; (2) perceived product quality and the likelihood that the product would be bought in the future; and (3) attitudes towards different bottles, including their appeal and user friendliness. They also read information about the different phases the study involved. The full study aim was revealed at the end of the study. Baseline assessments were conducted during this stage.

#### Stage 2: Run-in period—1 week

Households received their choice of wine(s) in both 750-ml and 375-ml bottles, to store in their homes and consume freely. This period functioned to acquaint households with the idea that wine would be delivered to them over the course of the study and that it came in two-sized bottles. It also served to determine whether delivered amounts were adequate.

#### Stages 3 and 4: Intervention periods—2 weeks each (4 weeks total)

During the intervention periods, a researcher visited participating households to deliver the total volume of wine for the forthcoming fortnight. At the end of the first intervention period, all unopened and partially opened bottles of study wine were removed and replaced immediately with the new size of bottle, i.e. the change-over was instantaneous with no washout. Following the procedures of a study using a similar design [26, 27], at the end of each intervention fortnight, households were requested to pay for the wine they consumed, at the rate specified on the study wine list. To avoid any potential confounding impact of price on consumption with each bottle size, the amount they paid per litre was kept constant across the intervention periods. Although existing stocks and collections of wine were not removed from households due to practical reasons, household members were asked not to consume from these for the duration of the study.

#### Stage 5: End of study and debriefing

Following the final assessment session, household representatives were fully debriefed by the researcher on the study aims. The debriefing process included an explanation of the study’s scientific aim and the reasons for not revealing this at recruitment (i.e. that awareness of the intervention’s purpose was expected to influence the outcome), as well as information regarding the adverse consequences of excessive alcohol consumption. Household representatives were asked to provide written consent for their household’s collected data to be used, having been informed of the scientific aims of the study.

At the end of the study, households received £150 worth of shopping vouchers for completion of all intervention periods and follow-up assessments.

#### Stage 6: Qualitative component

At the end of the study, household representatives were interviewed about their experience of drinking wine from each of the two bottle sizes and taking part in the study. The interviews were to explore the acceptability of the study procedures, whether participants were conscious of the study’s primary aim and if so, whether they thought this knowledge influenced their household’s consumption of wine. Interviews were semi-structured and last approximately 30 min. They were recorded and sent for external transcription. Transcripts of the interviews were anonymised.

### Baseline assessments

Upon giving consent to participation, household representatives were requested to complete a questionnaire regarding their household’s demographic characteristics, including the number of adults and children living in their home, their age, gender, highest educational qualification and annual household income. They were also asked to indicate their household’s wine preferences (red, white, blend, country of origin etc.), how much wine per week their household usually consumed at home, as well as the total amount their household drank outside the home. At this time, they were also given the study wine list and asked to choose the wine(s) they wanted to receive for the duration of the study. They were given time to study the wine list and discuss it with their household members. Households were offered the opportunity to sample the wines before selecting those they purchased for the study duration.

During a 2-week baseline period, households were requested to consume wine as usual but keep all empty and partially full bottles, based on which consumption was estimated. If there were discrepancies between households’ self-reported typical weekly consumption and the amount consumed during baseline, the highest of the two was used to determine the amount to be delivered during the intervention periods.

### Follow-up assessments

During the intervention periods, households were requested to continue to keep all delivered wine bottles, regardless of whether the contents were consumed, partially consumed or not consumed. They were told that the reason for this was to accurately estimate how much they needed to pay, based on the exact amount consumed. They were also requested to retain bottles of any non-study-supplied wine consumed during the interventions periods. Previous research has demonstrated that the validity of using empty bottle count to measure consumption can be compromised by guests from outside the household drinking the study-supplied drinks [27]. To account for this possibility, at the end of each week, household representatives received a questionnaire via email asking them to estimate how much, if any, of their wine was drunk by guests (see the “Outcomes” section). They were also asked to estimate the amount of wine their household consumed in and outside the home during the preceding week and were asked whether any of the study-supplied wine was consumed out of the home.

To build credibility for the cover story, households were asked to rate their consumption experiences. Where applicable, they were asked to estimate whether their guests appeared to have enjoyed the wine. If bottles of non-study-supplied wine were returned, household representatives were requested to compare their experiences of drinking the study-supplied vs the non-study-supplied wine. Although previous research using a similar design and procedures found that the majority of participants believed the cover story [27], in order to determine whether participants in the present study were aware of the purpose of the intervention and of the study’s aim, at the final follow-up assessment, household representatives were requested to state what they thought the study was about.

### Outcomes

#### Feasibility outcomes

- Recruitment rates, assessed by calculating the proportion of households entering the study from households identified as eligible
- Number of households discontinuing participation at follow-ups
- Practical problems associated with the following (encountered problems were noted in a document on a case by case basis):
  - Delivering the intervention, e.g. problems arranging timely delivery visits, to ensure each intervention period was of equal duration
  - Assessment procedures
  - Collection of consumption-related data e.g. problems related to participants adhering to instructions to keep all wine bottles whether full, partially full or empty

- Awareness of the study aim, assessed through (i) questionnaire and (ii) qualitative interviews

#### Acceptability outcomes, assessed through qualitative interviews

- Acceptability of the following:
  - Interventions
  - Study procedures

#### Outcomes for estimating the sample size for the planned study

- In-home consumption of study wine (in millilitres): Volume of study-supplied wine consumed by the household during each of the fortnightly intervention periods, measured by recording the numbers of empty and remaining full bottles. The remaining volume of partly consumed bottles was weighed and converted to millilitres.
- In-home consumption of non-study wine (in millilitres): Volume of non-study wine consumed by the household during each of the fortnightly intervention periods, measured by recording the numbers of empty and remaining full bottles. The remaining volume of partly consumed bottles was weighed and converted to millilitres.
- Out-of-home wine consumption (in millilitres): Volume of wine consumed by household members outside the household during each of the fortnightly intervention periods, measured by self-report by asking household representatives to indicate the number of days out-of-the home consumption occurred, as well as the number of small (125 ml), medium (175 ml), or large glasses (250 ml) or full bottles (750 ml) consumed on each one of those days.
- Guest in-home consumption of study wine (in millilitres): Volume of study-supplied wine consumed by non-household members (guests) during each of the fortnightly intervention periods, measured by household representatives self-reporting the number of days guests drank from the study wine, as well as the number of small (125 ml), medium (175 ml), or large glasses (250 ml) or full bottles (750 ml) they had consumed on each one of those days.

#### Other outcomes

- Characteristics of participating households, assessed through a questionnaire:
  - Index of Multiple Deprivation scores (derived from postcodes)
  - Total household income
  - Household composition (number of adults; number of children)
  - Highest education qualification obtained by any person within the household
  - Gender of all household members
  - Age of all household members
  - Number of wine drinkers in each household

### Sample size

This study was designed as a feasibility and acceptability study to inform a future, large-scale study. Consequently, a formal power calculation was not required [28]. The specific sample size was selected based on available resources (i.e. staff and funding) and our previous experiences in conducting feasibility studies of a similar design, aiming to reduce similar uncertainties [27]. It was estimated, however, that nine households per group could detect an effect size of 0.75 (difference in means = 0.75, SD of differences = 1), given a 2-sided test with alpha = 5% and 80% power in a 2 × 2 cross-over design with no other effects.

### Data analysis

The main analysis of this study included descriptive statistics of feasibility and acceptability outcomes. To estimate possible effect sizes from which to power a future larger study, differences in consumption of study-supplied wine between the two intervention periods were also calculated, controlling for household consumption of non-study-supplied wine, including out-of-home consumption and excluding consumption of study-supplied wine by non-household members (i.e. guests). As this was a feasibility study, however, and was not powered to detect possible differences in consumption with the different bottle sizes, no formal statistical analyses were planned or conducted.

Analysis of the anonymised data gathered through the semi-structured interviews was conducted following the principles of the Framework method [29]. The Framework method is an increasingly popular approach in medical and health research. It involves a systematic and flexible approach to organising and analysing qualitative data and a method of addressing specific research questions [30]. Its defining feature is the matrix output: rows representing cases and columns representing emerging concepts (termed “codes”). This allows the researcher to analyse the data in order both by case and by code [31]. It also allows in depth exploration of the data, while simultaneously maintaining an effective and transparent audit trail, which reinforces the rigour of the analytical processes and the credibility of the findings [31].

## Results

### Feasibility

#### Feasibility of recruitment and retaining eligible participants

**Fig. 1:**
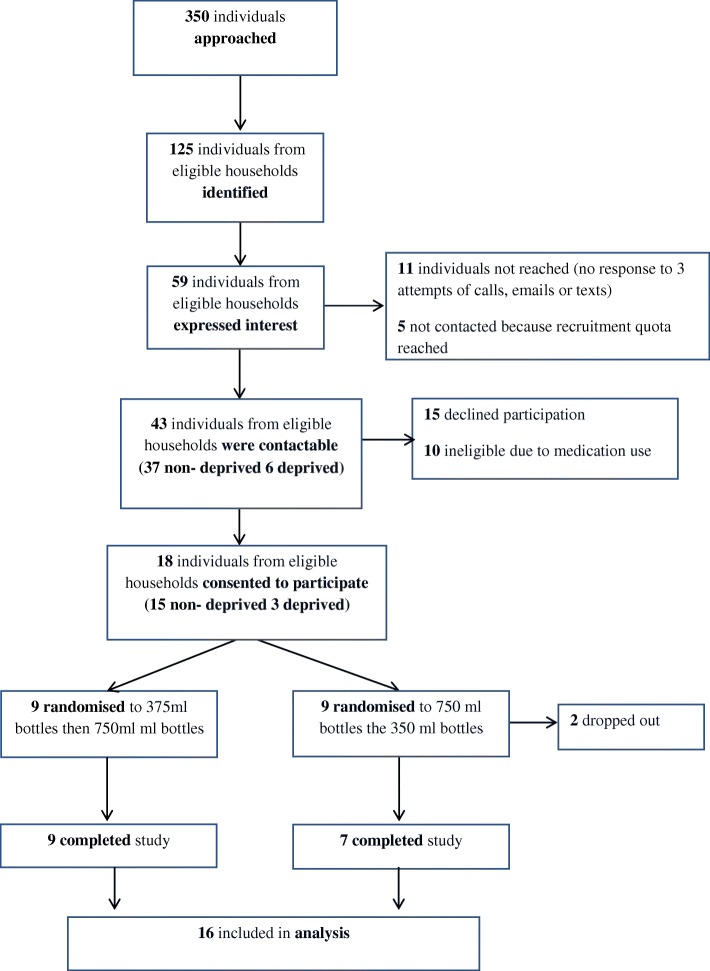
Flow of participants through study

Of the 350 individuals approached, 125 (36%) were from eligible households, of whom 58 (46%) expressed an interest in the study, 18 (14%) consented to take part and were randomised and 16 (13%) completed the study. Attrition between consenting to take part and completion of the study was 11% (2/18). No households dropped out between completion of the run-in phase and the first intervention period or between the two intervention periods (Fig. 1).

The majority (94%) of recruited households (*n* = 36) consisted of families with children and had a mean of 3.9 members (SD = 1.2; range= 2–6). The mean number of children per household was 1.7 (SD = 1.1.; range= 0–4), with a mean age of 9.5 years (SD = 5.4). The mean number of adult wine drinkers in each household was 1.7 (SD = 0.6; range= 1–3) and the mean adult age was 40.2 years (SD = 6.7). Just over half of all household members were female (54%). The educational level (as assessed by the highest educational qualification received by anyone in a household) of the majority of recruited households was classified as higher (i.e. beyond A levels or equivalent) (53%) and their annual income was classified as higher (beyond £25K) (88%). Based on area-level deprivation (Index of Multiple Deprivation scores) the majority (94%) of households were classified as not deprived. There were no significant differences between households randomised to the different bottle sizes during each intervention period in any of the above characteristics.

#### Feasibility of delivering intervention and assessment procedures

No major problems were reported with the study procedures, including delivery of the intervention and the assessment procedures.

One household made appointment time changes that affected the duration of their run-in period but only by a few hours. A further household reported having food poisoning during the second half of their intervention period, which influenced the amount of wine drunk.

#### Feasibility of collecting consumption-related data

All households returned all their wine bottles on all study weeks. Four households (25%) self-reported having guests who drank an estimated 3425 ml of the study wine (17% of the total amount delivered to all households) over 6 of the 64 household × intervention weeks. Ten households also reported drinking non-study wine outside the home on 19 of the 64 household × intervention weeks. The total self-reported wine consumed outside the home across all households was 18 l. No households reported drinking non-study wine while at home.

#### Credibility of cover story

Thirteen percent of households (2/16) guessed the study aim but did not guess the expected direction of effect, i.e. that consumption would be less with the smaller bottles. When interviewed about their experience of taking part in the study, those who guessed the aim reported that this knowledge had not affected their consumption with each bottle size. Those who did not guess the aim also reported that having such knowledge would not have affected their behaviour towards each bottle size (see the “Acceptability” section for relevant quotes).

### Acceptability

#### Acceptability of the intervention and study procedures

The study procedures, assessments and intervention were considered acceptable and no problems were reported by participating households. Participants expressed positive attitudes towards the study (“I think it was an interesting study … *.* Yeah, I was happy to participate” (Household 6); “It was really good, I thought it was a very well designed study” (Household 7)) and general procedures (“I didn’t find it particularly inconvenient, I didn’t, you know, it wasn’t a problem you coming round and, you know, doing the questionnaires and yeah, it was absolutely fine. Probably easier than I thought because I was a bit, you know, obviously it’s quite hard to know right at the beginning what to expect, like what to actually expect” (Household 15); “… everything’s been fine, communication has been good, lots of study, lots of information, lots of reassurance, not that I needed any! Yeah, your communication has been good, nice to get a text message just to remind me although you are on the calendar. No, it’s all been, it’s all been fine…” (Household 16)) and described the convenience of having the wine delivered to them ( “… it was really handy that you came out all this way to bring the wine, I felt a bit guilty about that” (Household 14)).

Minor issues were reported with regard to the wines participants had to choose from, namely their price (“The only thing I would say with the wine was a lot of it was expensive and I’d never ever pay that much for wine. (Household 1)) and the fact that they had to stick to their chosen wines for the duration of the study (“It would have been good, in the study, to actually be allowed to swap the wines that we ordered” (Household 4)). The use of a cover story was met with understanding and did not evoke any negative responses (“Um, no, I mean I understand the way you’ve got to create a story, I think that’s really important because... ...it would affect the results, definitely … No, I wasn’t annoyed. No, I wasn’t annoyed at all. I didn’t feel duped in any way and I now fully understand the reasons why you’ve got to create a story so that it doesn’t affect the results of the study” (Household 6); “I didn’t mind at all, I can see why you did it like that but yeah I mean I think it’s quite an important study actually, I felt it was quite good to be part of...” (Household 11)). Participants reported that having knowledge of the study’s real aims might have affected their overall drinking behaviour but not towards each bottle size (“I’d have probably tried to drink less! so as I didn’t look like an alcoholic (Household 2); “I just think I’d be aware of what I was drinking generally” (Household 9)).

### Sample size estimation

#### Possible effect size and variance of the intervention

Mean consumption across households with each bottle size—i.e. observed during each intervention period—with and without adjustments for self-reported guest and out-of-home consumption is shown in Table 1.

**Table 1:** Mean (SD) consumption in ml across households (*n* = 16) with each bottle size (each used for 2 weeks) with and without self-reported guest consumption and out-of-home consumption and according to intervention order

	Bottle size						Overall**	
	750 ml			375 ml				
	750 ml first (*n* = 7)	375 ml first (*n* = 9)	Overall* (*n* = 16)	750 ml first (*n* = 7)	375 ml first (*n* = 9)	Overall* (*n* = 16)	750 ml first (*n* = 7)	375 ml first (*n* = 9)
Excluding guest consumption and including out-of-home consumption	3371.3 (2198.5)	3396.0 (1780.4)	3385.2 (1903.7)	3964.1 (1698.5)	2919.9 (1685.4)	3376.7 (1719.0)	7335.4 (3732.9)	6315.9 (3293.5)
Excluding guest consumption and excluding out-of-home consumption	3082.0 (1813.9)	2590.4 (1157.7)	2805.5 (1447.2)	3346.3 (1264.7)	2431.0 (1483.1)	2831.4 (1425.8)	6428.3 (2981.9)	5021.4 (2518.1)
Including guest consumption and excluding out-of-home consumption	3332.0 (1490.6)	2618.2 (1182.8)	2930.5 (1329.9)	3417.7 (1267.5)	2533.8 (1365.8)	2920.5 (1357.4)	6749.7 (2657.4)	5152 (2389.4)

*Regardless of intervention order

**Regardless of bottle size

**Fig. 2:**
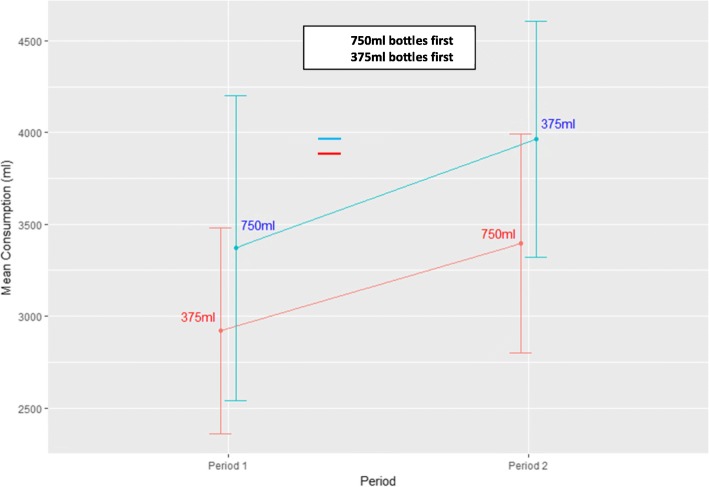
Mean consumption (ml), excluding guest and including out-of-home consumption, during each study period according to intervention order (± 1 SE)

After excluding guest consumption and including out-of-home consumption, consumption with the 750-ml bottles was greater compared to consumption with the 375-ml bottles by 8.4 ml (SD = 1235.4 ml; 95%CI [− 596.9, 613.8]) (750-ml bottles: 3385.2 ml (SD = 1698.5); 375-ml bottles: 3376.7 ml (SD = 1719.0 ml) Table 1). Results, however, suggest there may be an effect of ordering, with households that received the 375-ml bottles first consuming more wine out of the 750-ml bottles than from 375-ml bottles (750 ml consumption: 2618.2 ml (SD = 1182.8) vs 375 ml: 2533.8 ml (SD = 1365.8)) and vice versa (750-ml bottles first: 750 ml consumption: 3332.0 ml (SD = 1490.6) vs 375 ml: 3417.7 ml (SD = 1267.5)). This result might also reflect average consumption increasing with study duration regardless of bottle size, observed in the second intervention period (Table 1, Fig. 2). Furthermore, on average, less wine was consumed overall from both types of bottle when the 375-ml bottles were presented first, compared to the 750-ml bottles (375 ml first: 6315.9 ml (SD = 3293.5); 750 ml first: 7335.4 ml (SD = 3735.4)), even though the mean amount of wine delivered to each group was not significantly different (375 first: 4166.67 ml (SD = 847.91); 750 ml first: 4178.57 (SD = 1047.957)).

The effect of the intervention on consumption appears unclear for a number of reasons. These include the potential order effect and/or increase in consumption with study duration observed during the second half of the study, the different sample sizes for those receiving the 375-ml bottles first (*n* = 9) and those receiving the 750-ml bottles first (*n* = 7) and the small sample of the study. The sample size calculations for future studies are therefore estimated using the variance observed in the current study and a meaningful difference of 250 ml less wine consumed with the smaller bottles. This difference is based on each wine drinker per household drinking one fewer small wine glass (125 ml) per fortnight and an average of two wine drinkers per household (rounded up to the nearest whole person), observed in the current study.

Based on a difference of 250 ml in consumption and the variance of differences (i.e. SD = 1235.4 ml) suggested by the results relating to overall difference in consumption between the two bottle sizes (i.e. 8.4 ml), it is estimated that approximately 77 households per sequence (154 in total) would be needed for a future cross-over randomised study at 80% power and alpha of 5% [32].

## Discussion

This study aimed to assess the feasibility and acceptability of presenting a fixed volume of wine in different bottle sizes as a possible intervention for reducing in-home wine consumption and inform the design of a future large-scale randomised study. The study provides evidence for the feasibility of identifying, recruiting, and retaining eligible households and supports the feasibility of the study procedures, including delivery of the intervention and measurement of consumption. Furthermore, the results confirm the acceptability of the study procedures. The findings provide information relating to the variance of the intervention and thus inform the sample size calculations for the planned study.

One of the key uncertainties addressed in the present study was the feasibility of recruiting and retaining eligible participants, as well as estimating the required sample size for the future study. The recruitment rate achieved in the present study was above average for trials in the field of public health [33, 34], while study completion rates were comparable to previous research in the area [27, 33]. The effect size of the intervention on consumption was unclear given observed order and/or period effects and an imbalance between groups, among other issues. However, using a meaningful difference approach and the variance of the intervention suggested by the current results allowed estimation of the required sample size for a future cross-over randomised study; although caution is required regarding the precision of the estimate, given the small sample of the current study. To achieve the estimated sample size of 154 households, a minimum of 3370 households would need to be approached. This would require expanding the recruitment area beyond one city, to multiple locations, which would potentially increase the representativeness of the sample, which in the present study was limited.

Another uncertainty addressed by the present study was the feasibility of measuring wine consumption by recording the numbers of empty and remaining full bottles. The measure was found to be feasible, as all households adhered to the instructions to keep all bottles, whether full, partially full or empty, thus allowing estimation of consumption from leftover amounts in bottles. In previous research, the validity of this measure was compromised by guests from outside the household consuming from the study bottles [27]. In the present study, this occurred only on a minority of study weeks, possibly due to the type of drink being targeted. Previous research focused on sugar-sweetened beverages, consumption of which by guests might be more likely than wine, as they are arguably largely exempt from restrictions that might apply to alcoholic drinks (e.g. relating to the time of the day, the duration of the visit and activities planned for after the visit). The present study also included a measure of guest consumption, not included in previous research, which allowed estimation of consumption by household members only. A further factor undermining the validity of the measure of consumption in previous research was the failure to capture out-of-home consumption [27]. Although the present study included such a measure, the findings did not reveal any pattern of differential out-of-home wine consumption with the two bottle sizes. This difference could again be attributed to the type of drink assessed. It seems unlikely that smaller bottles of wine would be carried around and consumed out of the home, in the way smaller bottles of sugary drinks have been shown to be, thus potentially affecting out-of-home consumption with the different bottle sizes [27, 35].

Although the measure of consumption used in the present study was feasible, it will likely need to be adapted for use in future larger studies. Bottles were collected and assessed by a researcher visiting participants in their homes, a procedure permitted by the small sample size of the present feasibility study and the fact that all households were located within one city in England. The estimated sample size for the future study would require expanding the recruitment area to multiple locations, making in-person visits potentially challenging. One way to overcome this would be to conduct the study remotely (e.g. online and through the post) and rely on digital photographs of wine bottles to estimate amounts consumed, a method increasingly being used to accurately measure food and drink intake [29].

The study was not powered to detect differences in consumption with the different bottle sizes. Results, however, suggest a possible order effect, with consumption of wine being potentially less when the smaller bottles were presented prior to the standard bottles and more when smaller bottles were presented after the standard bottles. Given the small scale of this study—in addition to attrition and an unequal number of households receiving each of the two bottle sizes during each intervention period—it is not clear whether this effect represents a robust phenomenon. Indeed, this might also reflect consumption increasing with study duration regardless of bottle size—perhaps if households felt less inhibited about consumption at this point in the study. Further large-scale studies are needed to assess the impact of smaller bottles on alcohol consumption and elucidate the circumstances under which they might decrease or increase consumption. Smaller bottles might decrease consumption by making additional intake of wine more effortful, [10] or as a result of individuals’ tendency to consume a specific number of bottles in any one episode of consumption regardless of bottle size [20]. They might, however, also increase consumption by reducing barriers to consumption that are present for larger sizes [21] or as a result of being considered too small. The latter might be especially true, given that 750-ml bottles have become the standard size for wine internationally, which could distort perceptions of appropriate portion size with the smaller bottles. Indeed, exposure to larger portion sizes may alter perceptions of what constitutes a “normal”-sized portion [36]. Judgements of appropriate portion sizes can be especially hard for liquids and products made up of multiple units [37, 38]. At the time the current study was conducted, 375-ml bottles were available by some retailers in the UK. Since conducting the study, a limited range of non-premium wines has started to become available in 500-ml bottles in supermarkets in the UK [18]. This size—constituting two thirds of the standard wine bottle size—could potentially overcome any perceptions of being too small, as might be the case for 375-ml bottles. As such, 500-ml bottles might be more effective for decreasing wine consumption. The impact of various bottle sizes, including of 500-ml bottles and their relative effect to smaller bottles such as 375-ml bottles, should be examined in future research.

The impact of bottle size on alcohol consumption is likely to depend on other contextual factors, including the size of glasses in which alcohol is served and poured [13, 39]. Future research should focus on the combined effect of bottle size and glass size on consumption. The potential impact of bottle sizes smaller than 750 ml will be limited by their affordability, availability and acceptability, alongside alcohol and marketing strategies promoting their purchase [6–8, 40, 41]. If smaller bottles are shown to be effective in decreasing alcohol consumption, research and policy efforts should focus on determining ways to encourage their purchasing, for example by increasing the availability of non-premium wines in smaller bottles, placing them in areas of high visibility in retail stores and increasing their affordability relative to that of 75-cl bottles.

In conclusion, the findings of this study support the feasibility and acceptability of running a planned large-scale randomised study to assess the impact of bottle size on wine consumption at home. The results also provide information relating to the variance of the intervention needed to estimate the required sample size for such a study. The planned study is expected to provide the best estimate to date of the impact of smaller bottles on in-home wine consumption. This could thereby inform policy concerning the use of interventions targeting the size of products and their integral packaging to reduce alcohol consumption to improve population health.

## Data Availability

Available upon request
